# Metagenomic sequencing of mpox virus clade Ib lesions identifies possible bacterial and viral co-infections in hospitalized patients in eastern DRC

**DOI:** 10.1128/spectrum.00512-25

**Published:** 2025-05-30

**Authors:** Leonard Schuele, Leandre Murhula Masirika, Hayley Cassidy, Philip T. L. C. Clausen, Luca M. Zaeck, Marjan Boter, Pacifique Ndishimye, Jean Claude Udahemuka, Rory D. de Vries, Saria Otani, Richard Molenkamp, David F. Nieuwenhuijse, Justin Bengehya Mbiribindi, Freddy Belesi Siangoli, Marion Koopmans, Frank M. Aarestrup, Bas B. Oude Munnink

**Affiliations:** 1 Department of Viroscience, Erasmus University Medical Center, Rotterdam, the Netherlands; 2 Centre de Recherche en Sciences Naturelles de Lwiro, South-Kivu, Bukavu, Democratic Republic of the Congo; 3 Congo Outbreaks, Research for Development (CORD), South-Kivu, Bukavu, Democratic Republic of the Congo; 4 SaBio Instituto de Investigación en Recursos Cinegéticos IREC (Universidad de Castilla-La Mancha & CSIC) 552093 , Ciudad Real, Spain; 5 Research Group for Genomic Epidemiology, National Food Institute, Technical University of Denmark 5205, Lyngby, Denmark; 6 Stansile Research Organization, Kigali, Rwanda; 7 Research and Innovation Centre, African Institute for Mathematical Sciences (AIMS) 716610, Kigali, Rwanda; 8 Department of Veterinary Medicine, University of Rwanda 187195, Nyagatare, Rwanda; 9 Division Provinciale de la Santé, South-Kivu, Bukavu, Democratic Republic of the Congo; Children's National Hospital, George Washington University, Washington, DC, USA

**Keywords:** mpox, mpox virus, clade Ib, metagenomics, antimicrobial resistance, co-infections, monkeypox virus

## Abstract

Mpox is an emerging zoonotic disease that caused two public health emergencies of international concern within two years. Less is known about the interplay of microbial organisms in mpox lesions which could result in superinfections that exacerbate outcomes or delay recovery. We utilized a unified metagenomic sequencing approach involving slow-speed centrifugation and differential lysis on 19 mpox lesion swabs of hospitalized patients in South Kivu province (eastern DRC) to characterize bacteria, antimicrobial resistance genes, mpox virus (MPXV), and viral co-infections. High-quality MPXV whole-genome sequences were obtained until a Ct value of 27. Furthermore, co-infections with other clinically relevant viruses, such as varicella zoster virus and herpes simplex virus-2, were detected and confirmed by real-time PCR. In addition, metagenomic sequence analysis of the bacterial content showed the presence of bacteria associated with skin and soft tissue infection in 10 of the 19 samples analyzed. These bacteria had a high abundance of resistance genes, with possible implications for antimicrobial treatment based on the predicted antimicrobial resistance. In conclusion, we report the presence of bacterial and viral pathogens in mpox lesions and detection of widespread resistance genes to the standard antibiotic treatment. The possibility of a co-infection, including antimicrobial resistance, should be considered when discussing treatment options, along with the determination of the case-fatality ratio.

**IMPORTANCE:**

The mpox virus clade Ib lineage emerged in the eastern Democratic Republic of the Congo owing to continuous human-to-human transmission in a vulnerable patient population. A major challenge of this ongoing outbreak is its occurrence in regions with severely limited healthcare infrastructure. As a result, less is known about co-infections in affected patients. Identifying and characterizing pathogens, including their antimicrobial resistance, is crucial for reducing infection-related complications and improving antimicrobial stewardship. In this study, we applied a unified metagenomics approach to detect and characterize bacterial and viral co-infections in mpox lesions of hospitalized mpox patients in the eastern DRC.

## INTRODUCTION

Mpox virus (MPXV) is a double-stranded DNA virus of the Poxviridae family and the causative agent of mpox. It was first detected in humans in 1970, where it typically manifests with fever, lymphadenopathy, and vesiculopapular rash (1). MPXV is historically endemic to Central and Eastern Africa and genetically classified into two clades: clade I, associated with high case fatality rates (CFR), and clade II, which is thought to cause milder disease (2, 3).

Recently, we discovered a novel clade Ib MPXV virus that emerged in the eastern Democratic Republic of the Congo (DRC) (4). Clade Ib has since spread to neighboring countries in the east with continuous human-to-human transmission across the region (5). As of January 2025, clade Ib MPXV has been detected in Burundi, Tanzania, Rwanda, Uganda, Kenya, Zimbabwe, Zambia, Thailand, Sweden, Germany, France, Belgium, Canada, China, India, the UAE, the UK, and the USA (5). The continuous human-to-human transmission of MPXV in recent outbreaks seems to be primarily driven by sexual contact: while clade IIb transmission occurred almost entirely in men who have sex with men (MSM), clade Ib is mostly transmitted via (professional) heterosexual contact (6, 7). As a result, MPXV disproportionately affects MSM and professional sex workers (PSW), who represent a high-risk population for co-infection with sexually transmitted diseases (STDs) including HIV. Specifically in the DRC, an HIV seroprevalence of 7.5% and 0.7% has been reported in PSW and in the general population, respectively (8). In addition to sexual transmission, close direct contact (such as between caregivers and young children) has been observed (6).

The presence of microorganisms and their potential role in co- and secondary infections in mpox lesions is poorly studied, particularly outside of the global clade IIb outbreak occurring within the MSM community (9–12). Bacterial superinfection in mpox patients has been reported as a complication in the genital area and typically responds well to antibiotic treatment and is more prevalent among patients with HIV (9, 10, 13). Typically, mpox patients present with a skin rash with pox lesions (1). While clade IIb often affects a single anatomical location with few lesions, clade I pox lesions are more often found across the body (14). Pox lesions could be more prone to bacterial colonization and infection depending on the clinical context, microbial burden, and host immune response (9). Such co-infections could contribute to a higher morbidity or delayed recovery; however, many questions remain regarding how the presence of bacteria and/or other viruses affects the outcome, progression, and treatment of mpox cases (9, 15).

A major challenge is that the current mpox outbreak is occurring in regions with severely limited healthcare infrastructure, lacking even basic laboratory capacity to assess potential co-infections. A key question is how to integrate laboratory capacity into outbreak investigations. While investments are needed to build core laboratory infrastructure, they tend to focus on specific pathogens rather than broad detection capabilities. This is further emphasized by the trend of introducing point-of-care platforms, such as the Cepheid GeneXpert System, to enable rapid pathogen testing (16). However, point-of-care testing offers limited ability for pathogen characterization and flexibility to adapt to changing local needs (17). The identification and characterization of pathogens, including the determination of antimicrobial resistance, are crucial for reducing infection-related complications by improving antimicrobial stewardship. As metagenomic next-generation sequencing can detect and characterize all microorganisms in a sample, it is a promising tool to study microbial presence and diversity, along with potentially assisting the treatment of concomitant bacterial infections (18–20). Next-generation sequencing approaches have been evaluated and applied as first-line diagnostic testing under field conditions in low- and middle-income countries (18, 21). However, challenges still remain related to cost, limited accessibility in low-resource settings, long time to result, and difficult interpretation of the analytic output (20, 22).

In this study, we applied a unified metagenomic Nanopore sequencing approach on swabs obtained from mpox lesions caused by MPXV clade Ib from hospitalized patients in South Kivu, DRC, to assess its potential for the characterization of bacterial and viral pathogens.

## MATERIALS AND METHODS

### Sample selection

Ethical clearance was obtained from the Ethical Review Committee of the Catholic University of Bukavu (Number UCB/CIES/NC/022/2023). Lesion swabs were collected from the genital area of suspected mpox cases during admission and transferred into virus transport medium. Samples were collected from different health zones in South Kivu, including Kamituga, Miti-Murhesa, Nyangezi, Kadutu, and Mwenga. Samples were tested for MPXV using the GeneXpert Mpox assay (Cepheid) and stored at −20°C at the Provincial Health Division laboratory (DPS-Lab). A Material Transfer Agreement was approved for the research protocol of project UCB/CIES/NC/022/2023. Twenty positive samples with sufficient residual volume were randomly selected (collected between 12 August and 3 September 2024) and shipped on dry ice to the Erasmus MC for retrospective metagenomic sequencing to investigate potential co-infections.

### Sample pretreatment for metagenomic sequencing

We applied a unified metagenomics approach with depletion of human nucleic acids utilizing a combination of slow-speed centrifugation, saponin-based differential lysis, and osmotic pressure. Samples were centrifuged at 1,200 x g for 10 min; 150 µL of the supernatant was carefully removed and incubated at a total concentration of 0.2% saponin for 10 min at room temperature. Next, 150 µL of 50 mM Tris-HCl in water (pH 8) was added, mixed, and incubated for 30 sec, followed by the addition and immediate mixing of 3 µL NaCl (5M). Cell-free nucleic acids were then digested by adding Benzonase (250 U) (Merck) and MgCl_2_ to a concentration of 2.5 mM, followed by shaking at 600 rpm for 30 min at 37°C. Nucleic acids were extracted using the High Pure Viral Nucleic Acid Extraction Kit (Roche) without adding the poly(A) carrier RNA.

### Nucleic acid extraction and sequencing

Nucleic acids were amplified using a sequence-independent single-primer amplification (SISPA) method for random amplification (23, 24). Sequencing libraries were prepared with 100 ng of the product from each sample using the Native Barcoding Kit 24 v14 (Oxford Nanopore Technologies). Nineteen samples and a no-template control were run on a FLO-PRO114M flow cell (ONT) on a PromethION (ONT).

### Sequence analysis

Reads were basecalled with super-accurate basecalling and demultiplexed (barcode both ends, mid-read barcode filtering, trim barcodes) using Dorado Basecall Server 7.4.12 (ONT). SISPA primer sequences were removed using cutadapt v3.0 (25). Read QC metrics from the trimmed data were obtained using fastq v1.18.0 (https://github.com/mcollina/fastq). Human sequences were filtered out using minimap2 v2.28 (26). KrakenTools v1.2 was used for superkingdom and alpha diversity analysis using the Shannon index (27). Viral co-detections were confirmed with an NCBI blastn search.

Generation of viral consensus sequences, QC, clade assignment, and phylogenetic analysis were performed as previously described (6, 28). MPXV clade Ib reference sequences used for phylogenetic analyses were obtained from GISAID (acknowledged in Table S1) and GenBank. APOBEC3-style mutations were screened using Squirrel v1.0.12 (29), and the F13/VP37 gene was analyzed for tecovirimat resistance (30). The Papillomavirus Genotyping Tool v0.1 was used for the typing of papillomaviruses (https://www.rivm.nl/mpf/typingtool/papillomavirus/).

Bacterial species composition was determined by aligning reads against the KmerFinder database (downloaded 2024/10/01 and reindexed with prefix TG) using KMA v1.4.17 with the options -ont −1 t1 -mem_mode -ef (31). SNP distances were calculated between similar strains from different samples using KMA v1.4.17 with the -mint3 option and CCPhylo dist v0.8.5 with the option -P 10 (32). Antimicrobial resistance genes (ARGs) were identified by aligning reads against the ResFinder and LRE-Finder databases (2023/09/21, commit: 8117fca and 2024/03/05, commit fac445d, respectively) using KMA v1.4.17 with the options -ont -ef (31, 33, 34).

Prediction of phenotypic bacterial antimicrobial susceptibility from genotypes was based on a combination of the bacterial species in question and the presence of different ARGs. We based intrinsic and species-specific resistance on Murray (35) and phenotypic resistance encoded by the identified ARGs on the ResFinder database (33).

### Confirmation of viral co-infections by real-time PCR

Real-time PCR was performed for generic MPXV and for clade determination (36, 37). Real-time PCR was also performed for varicella zoster virus (VZV), herpes simplex virus type 1 (HSV-1), and herpes simplex virus type 2 (HSV-2) (38–41).

## RESULTS

### Sample cohort and sequencing

A total of 19 MPXV lesion samples which were collected from 19 hospitalized patients between 12 August and 3 September 2024 were randomly selected for metagenomic sequencing. Samples were collected from hospitalized patients from Kamituga (*n* = 10), Miti Murhesa (*n* = 5), Nyangezi (*n* = 2), Kadutu (*n* = 1), and Mwenga (*n* = 1) health zones. The median age of the patients was 22 years (6 years–40 years). Four patients were minors younger than 18 years, twelve were between 18 and 30 years, and three were between 31 and 45 years. Nine samples were collected from male patients and ten from female patients. Of the 15 adults above 17 years of age, six were working in the mining industry, six were PSWs, two were students, and one was a teacher. There were no reported deaths between the admission and release of the patient group.

A total of 46.98 Gbp of trimmed sequencing output was generated, with an average of 2.47 Gbp per sample (Table S2). On average, 20.13% of the reads were assigned to bacteria, 29.64% to viruses, and 36.36% to the host (Table S2). Alpha diversity ranged from 0.18 to 3.91, with an average of 1.03.

### Detection and characterization of bacteria

**Fig 1:**
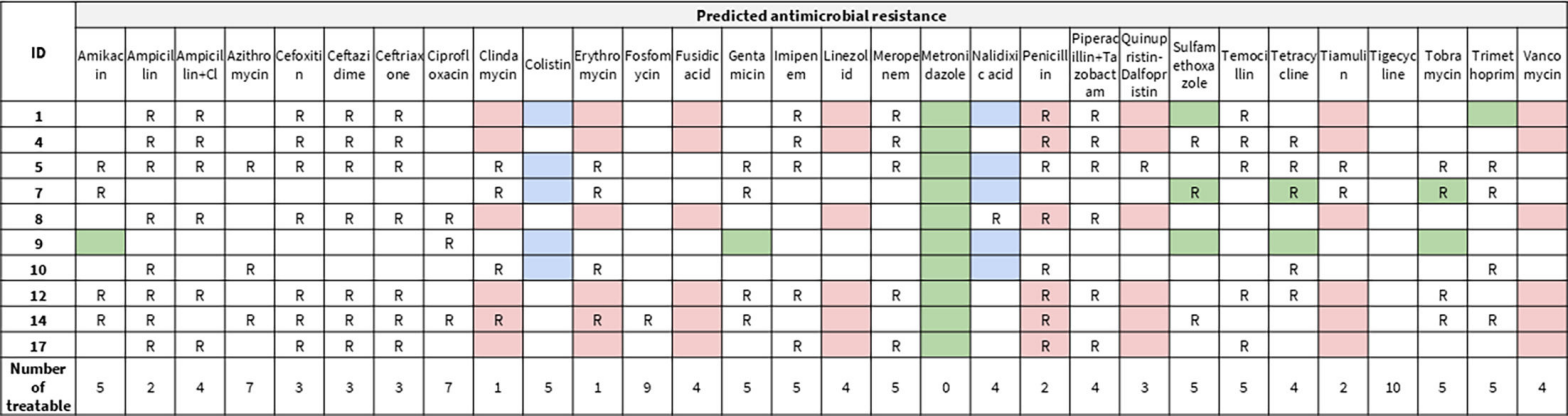
Predicted susceptibility based on the bacterial species and antimicrobial resistance genes present in the samples. RED: intrinsic resistance for gram-negative bacteria; BLUE: intrinsic resistance for gram-positive bacteria; GREEN: other species-specific intrinsic resistance.

Bacterial species with high genomic coverage (>90%) and depth (>3.0 x) could be identified in 10 of the 19 samples (Table 1). In some cases, only the genera could be assigned as the reads mapped to different species. In all cases, the identified species constituted more than 51% of the bacterial assigned bases and have been previously associated with skin and soft-tissue infection (SSTI). In samples 12 and 17, almost pure cultures of *Elizabethkingia meningoseptica* were identified with near complete genome coverage (97.98% and 97.93%) and a depth of coverage above 543 x and 65 x, respectively. SNP analysis revealed that the sequences were separated by 12 SNPs. The other most commonly observed bacterial species were *Enterococcus faecalis* (three samples), *Shewanella seohaensis* (two samples), *Klebsiella* sp. (two samples), *Staphylococcus aureus* (one sample), *Staphylococcus haemolyticus* (one sample), *Streptococcus pyogenes* (one sample), *Streptococcus dysgalactiae* (one sample), *Staphylococcus sciuri* (one sample), *Gardnerella* sp. (one sample), *Pseudomonas* sp. (one sample), and *Serratia* sp. (one sample). The *S. aureus* isolate could be assigned to sequence type (ST) 5 with a single SNP difference and the *S. haemolyticus* isolate to ST144 with a single SNP difference, while one *E. faecalis* could be assigned to ST16. A total of 45 different ARGs were identified in the 10 samples (between 2 and 17 per sample), with a breadth of coverage above 90%, depth of coverage above 3.0 x, and at least half the depth of the species detected in the corresponding sample (Table 1 and Fig. 1).

**TABLE 1:** Detected bacteria and linked antimicrobial resistance genes

ID	Bacteria	Antimicrobial resistance genes^*a*^
1	*Enterococcus faecalis* and *Shewanella seohaensis*	blaOXA-436*, blaCARB-2, and catA2*
4	*Shewanella* spp. (possibly seohaensis) / *Pseudomonas* spp. (possibly sediminis)	blaOXA-436*, blaCARB-2, catA2*, sul1*, and tet(M)*
5	*Enterococcus faecalis, Staphylococcus sciuri, and Macrococcus caseolyticus*	aac(6')-aph(2'')*, ant(6)-Ia, mecA, erm(B)*, sal(A)*, fexA, tet(K)*, tet(S)*, and dfrE
7	*Enterococcus faecalis and Staphylococcus haemolyticus*	aac(6')-aph(2''), ant(6)-Ia*, aph(3')-III, erm(B), erm(C), lsa(A)*, cat*, cat(pC221)*, tet(M), tet(K)*, and dfrG
8	*Klebsiella* spp. (possibly michiganensis or oxytoca)	aph(3')-Ia*, blaOXY-1–4*, and OqxB*
9	*Gardnerella* spp. (possibly piotii)	aph(3')-Ia and OqxB*
10	*Staphylococcus aureus* (main), *Streptococcus dysgalactiae,* and *Streptococcus pyogenes*	blaZ*, erm(C), tet(M), tet(K), dfrG
12	*Elizabethkingia meningoseptica*	aac(6')-Ij*, blaOXA-420, blaGOB-17, blaB-12, and tet(39)
14	*Klebsiella pneumoniae* and Serratia sp.	aph(3')-Ia, aac(6')-Ib-cr*, aac(3)-IIa, blaSRT-2*, blaOXA-1*, blaTEM-1C, blaZ_78*, blaCTX-M-15, fosA6*, mef(A)_2*, catB3*, catA1*, qnrS1, OqxB*, sul1*, sul2*, and dfrA15
17	*Elizabethkingia meningoseptica*	blaGOB-17 and blaB-12

^*a*^ “*” indicates an imperfect match.

### Detection and characterization of viruses

**Fig 2:**
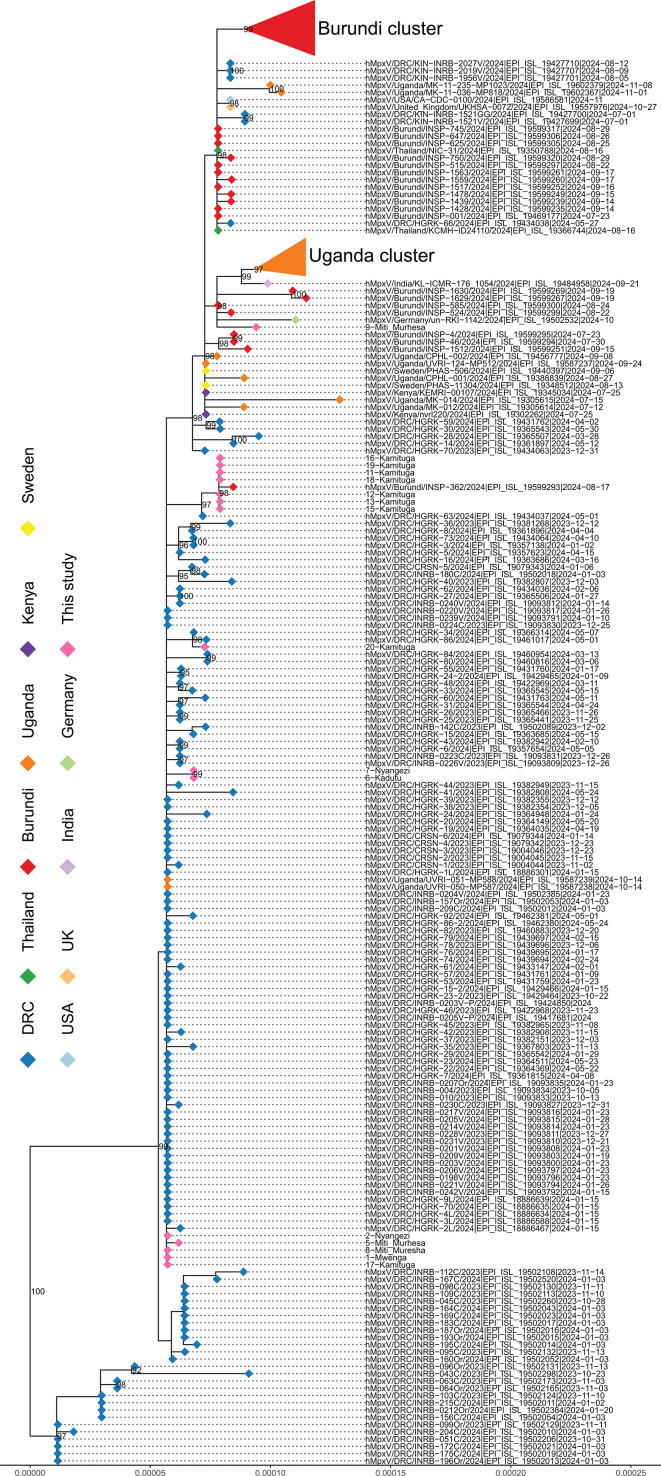
Phylogenetic analysis of clade Ib mpox virus sequences from this study (highlighted in pink) and publicly available clade Ib sequences.

We detected MPXV in 16/19 samples using metagenomic sequencing, while real-time PCR identified MPXV in 17/19 samples. All MPXV sequences were classified as clade Ib. The Ct values of the samples ranged from 11.1 to 32.8, with an average of 18.6 (Table 2). We obtained near-complete (≥ 98.5%) MPXV sequences from 16 of the 19 samples. No mutations related to tecovirimat resistance were detected in the F13/VP37 gene. Most mutations were APOBEC3-style mutations. Phylogenetic analysis of the 16 MPXV sequences showed minor variation between the sequences. The sequences consisted of mainly two clusters, clustering with other publicly available MPXV sequences from South Kivu collected in 2024 (Fig. 2). One cluster of seven sequences from this study clustered with a sequence from the bordering country of Burundi, collected during the same time period.

**TABLE 2:** Demographics, MPXV Ct values, MPXV sequence metrics, viral detections, and presence of bacteria associated with skin and soft-tissue infections^*b*^

ID	Gender	Age^*a*^	Ct MPXV	MPXV coverage (> 95%)	MPXV depth	MPXV total bases	Viral detections	Bacteria linked to SSTI
1	Male	Adult	25.9	98.5%	42.4 x	0.6%	MPXV and TTV	Yes
2	Male	Minor	16.7	100%	8325.6 x	88.7%	MPXV and TTV	
4	Male	Adult	ND	ND	ND	ND	TTV	Yes
5	Female	Minor	14.3	100%	13209.4 x	93.5%	MPXV and TTV	Yes
6	Female	Minor	16.3	100%	8052.9 x	84.2%	MPXV and TTV	
7	Male	Adult	17.0	100%	5675.3 x	46.4%	MPXV and TTV	Yes
8	Female	Adult	11.1	100%	10654.4 x	93.3%	MPXV and TTV	Yes
9	Female	Adult	22.5	100%	11478.7 x	74.3%	MPXV, TTV, VZV (Ct 31.9, 4.4% genome coverage), HPV-175, and HBoV-1	Yes
10	Female	Minor	ND	ND	ND	ND	TTV and VZV (Ct 29.5, 15.5% genome coverage)	Yes
11	Male	Adult	17.4	99.9%	70.1 x	6.3%	MPXV and TTV	
12	Female	Adult	17.7	99.8%	420.3 x	5.2%	MPXV and TTV	Yes
13	Male	Adult	16.6	99.8%	205.0 x	2.4%	MPXV and TTV	
14	Male	Adult	32.8	ND	ND	ND	MPXV and TTV	Yes
15	Male	Adult	16.6	99.9%	1372.7 x	12.1%	MPXV and TTV	
16	Female	Adult	15.8	100.0%	1113.0 x	11.1%	MPXV and TTV	
17	Female	Adult	27.2	98.5%	55.1 x	0.4%	MPXV and TTV	Yes
18	Female	Adult	16.5	100.0%	1998.0 x	15.2%	MPXV and TTV	
19	Female	Adult	17.5	99.9%	481.2 x	3.2%	MPXV and TTV	
20	Female	Adult	14.9	100.0%	6524.4 x	56.1%	MPXV, TTV, and HSV2 (Ct 27.0, 0.3% genome coverage)	

^*a*^ Minors (0–17 years); adults (>17 years). Sample 3 had insufficient sample volume for metagenomic sequencing.

^*b*^ Abbreviations: SSTI, Skin and soft-tissue infections; TTV, torque teno virus; VZV, varicella zoster virus; HPV, human papillomavirus; HSV-2, herpes simplex virus-2; HBoV-1, human bocavirus 1; MPXV, mpox virus; Ct, cycle threshold; ND, not detected. Sample 3 had insufficient sample volume for metagenomic sequencing.

### Co-detections

In 9 of the 10 samples with bacteria associated with skin and soft-tissue infections, a viral pathogen was co-detected (Table 2). In one sample, only bacteria associated with SSTIs were detected, while in the remaining nine samples, only a viral pathogen was detected. Two patients appeared to have co-infections with other viruses, along with MPXV. In patient 9, VZV, human papillomavirus 175, and human bocavirus 1 were all detected in a PSW, while in patient 20, herpes simplex virus type 2 (HSV-2) was detected in a PSW. Furthermore, VZV was also detected in a minor. As only small fractions of the genomes of VZV were obtained, no further characterization was performed as VZV and HSV-2 variants occur mostly through SNPs. Human bocavirus-1 (21.6% genome coverage [1,210/5,591 bp]) was most similar to an isolate from a patient in Sri Lanka (PQ273469). Torque teno viruses (TTV) were detected in all 19 samples.

## DISCUSSION

MPXV is a zoonotic pathogen with increasing diversity and ongoing human-to-human transmission in different regions of the world. Metagenomic sequencing can enable the characterization of microbial organisms for genomic surveillance and potentially assist in guiding antimicrobial stewardship in cases of co-infection or concomitant infections. However, the untargeted nature of shotgun metagenomic sequencing can result in limited sensitivity in complex sample types or samples with high quantities of host nucleic acid background (22). Approaches to increase sensitivity include centrifugation, differential lysis, probe-based capture, and osmotic pressure (42–46). We reduced the human nucleic acid background by applying slow-speed centrifugation, saponin-based differential lysis, and osmotic pressure, which enabled the characterization of bacterial and viral pathogens in mpox lesion samples.

The bacterial information obtained from our metagenomic analysis is not proof that those bacteria were involved in infections. However, in the absence of any other clinical diagnostics, it does provide insights into which bacterial species could be associated with mpox lesions and thus contribute to disease severity. Interestingly, most of the sequence data could be assigned to a single or few species, which does indicate some localized multiplication of single species and not only the presence of contaminating mixed skin flora. All of the bacterial species identified have previously been associated with skin and soft-tissue infections. In a few cases, we were able to obtain sufficient data to determine the MLST and the identification of *S. sciuri* with a mecA gene, which is not surprising. Interestingly, the two sequences from samples with *E. meningoseptica* were identical but were associated with different ARGs.

The possibility to treat a bacterial infection depends on the intrinsic susceptibility of the bacterial species in question and any acquired resistance typically measured using phenotypic testing. In recent years, several studies have shown the potential of predicting phenotypes only based on genotypes and found good concordance, especially for some species (47–49). Using metagenomics, it is not possible to assign ARGs with certainty to individual bacterial species. However, in situations where any other clinical microbiological data are lacking, it can provide information on which ARGs might be present in those bacteria and thus potentially assist in guiding treatment. The antimicrobial treatment which would have been prescribed to the patients in all cases would be ceftriaxone, cloxacillin, and metronidazole [Murhula, personal communication]. Metronidazole is typically only active against anaerobic bacteria, whereas ceftriaxone and cloxacillin are typically used against bacteria resistant to simple beta-lactams. We would only expect ceftriaxone or cloxacillin to be effective against the bacteria identified in patients 7, 9, and 10. The predicted most efficient antimicrobials would be ciprofloxacin, fosfomycin, and tigecycline; however, these antibiotics are not currently routinely available in South Kivu. In addition, it should be noted that quinolone resistance often is caused by chromosomal point mutations, which were not identified in this study. Crucially, culturing of bacterial isolates followed by phenotypic resistance confirmation was not possible due to BSL-3 biosafety and sample volume limitations, representing a limitation of this study. Moreover, treatment, patient history, and clinical information were limited, preventing direct associations between bacterial detections and abundance with patient status and recovery. As this was a retrospective pilot study, future work should include swabs of suspected (negative) mpox samples, bacterial culturing, phenotypic antimicrobial susceptibility testing, and comprehensive patient follow-up.

With regard to viral detections, VZV was detected twice. The first case was in a patient with a confirmed MPXV detection, and the second case was in a patient with locally diagnosed MPXV that could not be confirmed with real-time PCR and sequencing at Erasmus MC. Infections with VZV have been reported to be challenging to differentiate from mpox (50, 51) and particularly in children from regions with mpox outbreaks, who are frequently exposed to both pathogens (52). HSV-2 and HPV-175 were also detected, both of which are common sexually transmitted diseases and therefore likely a reflection of the sampling location (6, 53). TTVs were detected in all samples of this study. TTV is considered part of the human virome and could possibly be an indicator for the immune status (54).

In conclusion, we show that metagenomic sequencing can enable the characterization of viral and bacterial pathogens in mpox lesion samples for genomic surveillance and can potentially help guide treatment options. While our study is only a starting point to assess the viral and bacterial diversity in mpox lesions, it highlights the need for further research to better understand the clinical significance and dynamic relationships between other pathogenic infections and emerging mpox viruses.

## Data Availability

Mpox virus consensus sequences have been deposited on GISAID under the accession IDs: EPI_ISL_19689371-19689381, EPI_ISL_19689399, EPI_ISL_19689575, EPI_ISL_19689632, and EPI_ISL_19689707. The sequencing reads have been deposited on the European Nucleotide Archive under project: PRJEB88042.

## SUPPLEMENTAL MATERIAL

Supplemental tables spectrum.00512-25-s0001.docx Tables S1 and S2.

ASM does not own the copyrights to Supplemental Material that may be linked to, or accessed through, an article. The authors have granted ASM a non-exclusive, world-wide license to publish the Supplemental Material files. Please contact the corresponding author directly for reuse.
