# Supplementary material for: Metagenomic sequencing of mpox virus clade Ib lesions identifies possible bacterial and viral co-infections in hospitalized patients in eastern DRC
Source: Microbiol Spectr. 2025 May 30;13(7):e00512-25. doi: 10.1128/spectrum.00512-25 (PMC12211058; doi:10.1128/spectrum.00512-25)
Supplement: Supplemental tables — Tables S1 and S2. [file spectrum.00512-25-s0001.docx]

Table S1. Acknowledgement for the phylogenetic tree background sequences from GISAID and supplemental GenBank accession numbers: PP601207.1 - PP601228.1.


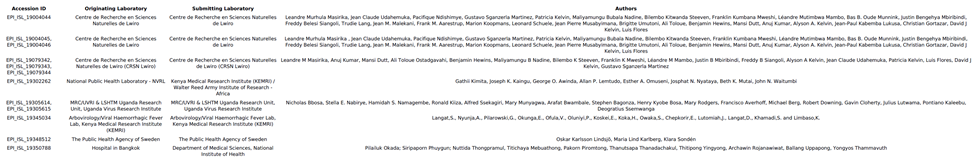


Table S2. Sequencing metrics, detection of SSTI associated bacteria, superkingdom (percentage of total reads per sample) and alpha diversity analysis of sequenced mpox lesions.

| **# ID** | **Output in Gbp** | **Mean length (bp)** | **SSTI bacteria & ARGs** | **Bacteria (%)** | **Viruses (%)** | **Host (%)** | **Archaea (%)** | **Fungal (%)** | **unclassified (%)** | **Alpha diversity** |
| --- | --- | --- | --- | --- | --- | --- | --- | --- | --- | --- |
| **1** | 1.99 | 827 | yes | 6.12 | 0.36 | 2.88 | 0 | 0 | 90.62 | 3.91 |
| **2** | 2.30 | 1,005 |  | 2.92 | 87.53 | 1.36 | 0 | 0 | 8.18 | 0.29 |
| **4** | 1.89 | 995 | yes | 82.5 | 0.02 | 3.58 | 0 | 0 | 13.87 | 3.08 |
| **5** | 2.46 | 871 | yes | 4.05 | 93.07 | 1.54 | 0 | 0 | 1.33 | 0.27 |
| **6** | 3.11 | 1,064 |  | 2.66 | 83.18 | 1.7 | 0 | 0 | 12.46 | 0.27 |
| **7** | 1.93 | 949 | yes | 6.24 | 43.44 | 2.2 | 0 | 0 | 48.1 | 0.78 |
| **8** | 2.78 | 703 | yes | 4.13 | 92.77 | 1.47 | 0 | 0 | 1.61 | 0.32 |
| **9** | 2.52 | 959 | yes | 8.85 | 71.18 | 4.62 | 0.01 | 0 | 15.3 | 0.70 |
| **10** | 2.84 | 991 | yes | 32.67 | 0.41 | 4.54 | 0 | 0 | 62.19 | 1.32 |
| **11** | 1.75 | 943 |  | 0.15 | 5.45 | 93.93 | 0 | 0 | 0.46 | 0.51 |
| **12** | 1.63 | 961 | yes | 31.2 | 4.38 | 63.22 | 0 | 0 | 1.19 | 1.73 |
| **13** | 2.01 | 885 |  | 0.25 | 2 | 97.29 | 0 | 0 | 0.45 | 1.05 |
| **14** | 5.08 | 1,004 | yes | 94.78 | 0.28 | 1.97 | 0 | 0 | 2.92 | 1.93 |
| **15** | 2.65 | 911 |  | 0.21 | 9.68 | 89.69 | 0 | 0 | 0.41 | 0.45 |
| **16** | 1.81 | 960 |  | 0.22 | 8.51 | 90.76 | 0 | 0 | 0.51 | 0.47 |
| **17** | 2.59 | 994 | yes | 97.2 | 0.42 | 1.1 | 0 | 0 | 1.28 | 0.21 |
| **18** | 3.02 | 828 |  | 7.84 | 12.39 | 79.16 | 0 | 0 | 0.59 | 1.27 |
| **19** | 2.29 | 885 |  | 0.15 | 2.32 | 97.06 | 0 | 0 | 0.47 | 0.87 |
| **20** | 2.34 | 892 |  | 0.36 | 45.86 | 52.77 | 0 | 0 | 1 | 0.18 |
| **Avg** | **2.47** | **928** | **10 out of 19** | **20.13** | **29.64** | **36.36** | **0.00** | **0.00** | **13.84** | **1.03** |

Sample 3 had insufficient remaining sample volume for metagenomic sequencing. **Abbreviations:** Avg, average; SSTI, skin and soft-tissue infection; Gbp, giga base pairs
